# Measurement of Serum Free Thyroxine Index May Provide Additional Case Detection Compared to Free Thyroxine in the Diagnosis of Central Hypothyroidism

**DOI:** 10.1155/2015/965191

**Published:** 2015-12-08

**Authors:** Kevin M. Pantalone, Betul Hatipoglu, Manjula K. Gupta, Laurence Kennedy, Amir H. Hamrahian

**Affiliations:** ^1^Endocrinology and Metabolism Institute, Cleveland Clinic, Desk F-20, 9500 Euclid Avenue, Cleveland, OH 44195, USA; ^2^Pathology and Laboratory Medicine Institute, Cleveland Clinic, Desk LL3-140, 9500 Euclid Avenue, Cleveland, OH 44195, USA; ^3^Department of Endocrinology, Cleveland Clinic Abu Dhabi, Abu Dhabi, UAE

## Abstract

The diagnosis of central hypothyroidism is often suspected in patients with hypothalamic/pituitary pathology, in the setting of low, normal, or even slightly elevated serum TSH and low free thyroxine (FT4). We present four cases of central hypothyroidism (three had known pituitary pathology) in whom central hypothyroidism was diagnosed after the serum free thyroxine index (FTI) was found to be low. All had normal range serum TSH and free thyroxine levels. This report illustrates that the assessment of the serum FTI may be helpful in making the diagnosis of central hypothyroidism in the appropriate clinical setting and when free T4 is in the low-normal range, particularly in patients with multiple anterior pituitary hormone deficiencies and/or with symptoms suggestive of hypothyroidism.

## 1. Background

Central hypothyroidism is a rare cause of hypothyroidism in the general population, estimated to occur in 1 : 20,000 to 1 : 80,000 [1]. However, in patients with pituitary pathology, it is observed much more frequently, particularly in patients with other pituitary hormone deficiencies. The diagnosis of central hypothyroidism is often suspected in patients with hypothalamic/pituitary pathology, in the setting of low free thyroxine (FT4). Most clinicians measure serum free thyroxine rather than total T4 or free thyroxine index to avoid problems with thyroid binding proteins. Pituitary mass lesions are the most common cause of central hypothyroidism. In cases of central hypothyroidism, serum TSH can be low, within the normal reference range, or even slightly elevated since TSH may have reduced biologic activity but normal immunoactivity [2–4]. In cases where FT4 is frankly low, making the diagnosis of central hypothyroidism is usually straightforward. However, in cases where free T4 is within the normal reference range, especially in the setting of normal serum TSH, making the diagnosis of central hypothyroidism can be challenging. We present four cases of central hypothyroidism that were diagnosed via the assessment of serum FTI, in cases where patients had serum TSH and FT4 values within their respective reference ranges.

## 2. Methods

A retrospective chart review was completed on four subjects seen in our pituitary clinics with suspected central hypothyroidism, with serum TSH and free T4 measures within their respective reference ranges, in the setting of low serum FTI. All of the reported laboratory tests were performed at our institution's reference laboratory.

Total T4 and T-uptake both were measured on Roche Elecsys electrochemiluminescence immunoassay analyzer. Total T4 is measured by competitive inhibition assay using specific T4 antibody labeled with ruthenium complex. Serum T4, released from binding proteins by the action of 8-anilino-1-naphthalene sulfonic acid, competes with the added biotinylated T4 for the binding sites on the antibodies. Percent coefficient of variation for interassay precision was <5%. The reference range for adults is 5–11 ng/dL.

Thyroxine binding capacity (TBC) is measured by T-uptake immunoassay. For this patient, serum is first incubated with exogenous T4 and biotinylated T4 polyhapten that binds to the free binding sites in the serum. Labeled T4 specific antibody (ruthenium) was added which complexes with the biotinylated T4 polyhapten. This antibody-T4 biotin complex is then separated by addition of streptavidin coated microparticles and chemiluminescence is measured which is inversely proportional to the exogenous T4 concentration. Results are generated by 2-point calibration curve and the analyzer automatically calculates the T-uptake as thyroxine binding index (TBI). Free thyroxine index is calculated by dividing the total T4 by the TBI value (T-uptake ratio). Interassay precision (CV) is <5%. Reference range for T-uptake ratio is 0.7–1.2 and for FTI is 6–11.00 *μ*g/dL.

Free T4 was also measured with the use of specific anti-T4 antibody labeled with a ruthenium complex which binds the free T4 in the serum in the first incubation. This is followed by the addition of biotinylated T4 that binds to the remaining free binding sites on the T4 antibody. The antibody complexes are then removed by addition of streptavidin coated microparticles and chemiluminescence is measured.

TSH was measured by two-site electrochemiluminescence immunoassay on cobas immunoassay analyzer from Roche Diagnostics. Lower limit of detection is 0.005 and interassay precision [CV] at two levels is <5%.

## 3. Results and Discussion

### 3.1. Case Reports

*Case 1.* A 55-year-old woman presented to endocrinology for management of type 2 diabetes (T2D). During her evaluation, multinodular goiter was appreciated on physical exam. A thyroid ultrasound noted a 5.9 × 4.4 × 4.0 cm complex nodule occupying the left lobe. Her TSH was 1.49 (0.4–5.5 *μ*U/mL). The nodule underwent fine-needle aspiration and was found to be benign. The patient reported chronic fatigue, weight gain, constipation, and hair thinning. Her TSH had been measured multiple times and always reported within the normal range. She asked for additional evaluation of her thyroid function, so her TSH was repeated and an assessment of free thyroxine (FT4) was conducted: TSH 1.06 (0.4–5.5 *μ*U/mL) and FT4 0.7 (0.7–1.8 ng/dL). The thyroid function tests were repeated in six weeks and were as follows: TSH 0.80 (0.4–5.5 *μ*U/mL), FT4 0.7 (0.7–1.8 ng/dL), and FT3 2.2 (1.8–4.6 pg/mL). Because the free thyroid hormone levels were on the lower side of the normal reference range, a complete assessment of pituitary function was performed and was notable for undetectable gonadotropins and a prolactin level of 1775.2 (2.0–17.4 ng/mL). An ACTH stimulation test demonstrated a normal cortisol response and serum IGF-1 was within normal limits. A 1.8 cm sellar mass (prolactinoma) was subsequently found on computed tomography (CT). Her thyroid function tests were repeated, this time with an assessment of serum FTI and total T4 and T3; the results were as follows: TSH 0.92 (0.4–5.5 *μ*U/mL), FT4 0.7 (0.7–1.8 ng/dL), FT3 2.1 (1.8–4.6 pg/mL), FTI 3.8 (6–11 *μ*g/dL), T4 4.0 (5.0–11 *μ*g/dL), and T3 88 (94–170 ng/dL). Cabergoline (0.5 mg twice per week) and levothyroxine 100 mcg daily were initiated and subsequently her prolactin and thyroid function tests after three months of therapy were as follows: prolactin 1.1 (2.0–17.4 ng/mL), FTI 9.2 (6–11 *μ*g/dL), free T4 1.4 (0.7−1.8 ng/dL), and TSH 0.466 (0.4–5.5 *μ*U/mL). The patient reported a significant improvement in her symptoms and has experienced a modest weight loss since the initiation of therapy.

*Case 2.* A 36-year-old male presented for a second opinion regarding the management of acromegaly. He underwent craniotomy for the management of a prolactin and GH secreting tumor six years ago. Subsequently, his prolactin and IGF-1 levels improved, but residual tumor and persistent hormone elevations were noted. He was initiated on octreotide LAR and bromocriptine; however, his tumor continued to increase in size. The second surgery was not successful to normalize IGF-1 and prolactin levels. During his evaluation, he reported fatigue and erectile dysfunction. He developed secondary hypogonadism and adrenal insufficiency after his initial surgery. During his initial hormone assessment postoperatively, he was noted to have slightly low TSH 0.32 (0.4–5.5 *μ*U/mL), on one occasion, but with a normal range serum FT4 1.1 (0.7–1.8 ng/dL). All subsequent TSH and FT4 assessments were within the normal reference range; levothyroxine therapy was never initiated. Repeat assessment of his thyroid function tests, at the time of his initial encounter at our institution, showed the following: TSH 0.85 (0.4–5.5 *μ*U/mL), FT4 0.9 (0.7–1.8 ng/dL), FT3 3.0 (1.8–4.6 pg/mL), FTI 4.9 (6–11 *μ*g/dL), T4 4.4 (5.0–11 *μ*g/dL), and T3 81 (94–170 ng/dL). Initiation of levothyroxine therapy resulted in improvement in his fatigue. Subsequent thyroid function tests, while taking 150 mcg of levothyroxine daily, were as follows: FTI 7.5 (6–11 *μ*g/dL), TSH 0.120 (0.4–5.5 *μ*U/mL), FT4 1.3 (0.7–1.8 ng/dL), and FT3 3.3 (1.8–4.6 pg/mL).

*Case 3.* A 60-year-old male presented with the complaints of blurry vision (left > right) and profound fatigue. Evaluation led to the discovery of a visual field deficit (left > right) and a large sellar mass (3.0 cm) with suprasellar extension and bilateral cavernous sinus invasion. Magnetic resonance imaging characteristics were suggestive of pituitary macroadenoma. His labs were consistent with secondary hypogonadism, but his remaining pituitary function tests were within their respective reference ranges: IGF-1 110 (60–211 ng/mL), random cortisol 20.9 *μ*g/dL, TSH 4.06 (0.4–5.5 *μ*U/mL), and FT4 0.7 (0.7–1.8 ng/dL). Given the notion that the FT4 level was on the lower side of the reference range and our experience with similar cases in the past, he underwent further assessment of his thyroid function: TSH 3.56 (0.4–5.5 *μ*U/mL), FT4 0.8 (0.7–1.8 ng/dL), FTI 4.2 (6–11 *μ*g/dL), T4 5.6 (5.0–11 *μ*g/dL), and T3 100 (94–170 ng/dL). Levothyroxine 50 mcg daily was initiated, and follow-up thyroid function tests were as follows: TSH 0.936 (0.4–5.5 *μ*U/mL) and FTI 7.7 (6–11 *μ*g/dL). He noted an improvement in his fatigue. The patient subsequently underwent subtotal surgical resection of the nonfunctional mass followed by gamma knife stereotactic radiosurgery. His visual field deficit improved. Repeat thyroid function tests, while taking 50 mcg of levothyroxine, 1 month and 5 months postoperatively, revealed slightly low TSH and midnormal range serum FTI. His levothyroxine was increased to 75 mcg.

*Case 4.* A 62-year-old male presented to an outside hospital emergency department with symptoms of stroke. The evaluation of his right-sided hemiparesis included a CT scan of the brain, which showed an incidental sellar mass (3.5 cm). MRI characteristics were consistent with macroadenoma. A full assessment of his pituitary function tests showed hypopituitarism: testosterone < 12 (220–1000 ng/dL), LH 2.0 (1.0–7.0 mU/mL), FSH 2.7 (1.0–10.0 mU/mL), IGF-1 13 (39–231 ng/mL), peak cortisol level after cosyntropin stimulation 6.7 mg/dL (>18 *μ*/dL), TSH 5.44 (0.4–5.5 *μ*U/mL), FT4 0.7 (0.7–1.8 ng/dL), FTI 4.3 (6–11 *μ*g/dL), T4 5.5 (5.0–11 *μ*g/dL), and prolactin 44.1 (2.0–14.0 ng/mL). He was initiated on hydrocortisone (20 mg per day) and levothyroxine therapy 75 mcg daily; subsequent thyroid function tests were as follows: TSH 3.6 (0.4–5.5 *μ*U/mL) and FTI 6.7 (6–11 *μ*g/dL). The patient underwent surgical resection of the mass; pathology was consistent with a nonfunctional pituitary adenoma.

Please see Table 1 for a complete summary of the lab data provided.

Our report highlights the utility of assessing serum FTI in making the diagnosis of central hypothyroidism, particularly when the serum TSH value is within the normal reference range and free T4 is in the lower part of the normal range. As it was the situation in the cases discussed, free T4 in the low-normal range failed to prompt the appropriate diagnosis and initiation of thyroxine in most cases. T3 values (total or free) are usually not helpful in making the diagnosis of central hypothyroidism, as both are frequently within the normal range in patients with mild hypothyroidism due to increased 5′-deiodinase activity [5–7]. Gupta et al. have previously reported that free T4 assessment is less sensitive for detecting hypothyroidism as compared to FTI [8]. In all four cases, the FTI was clearly low and was consistent with the presence of partial central hypothyroidism in the context of the clinical presentation and other pituitary function studies. Based on our experience over the years and in the appropriate clinical setting, we use serum FTI < 5 (6–11 *μ*g/dL) to diagnose central hypothyroidism.

In certain cases, a TRH stimulation test may be useful in the evaluation of suspected cases of central hypothyroidism. Baseline TSH is obtained, and then 200 mcg of TRH is administered as an IV bolus. TSH values are subsequently obtained at 20 and 60 minutes after TRH administration. The normal increment in TSH at 20 min is 5–30 (mean 15) IU/mL with slight diminution at 60 min (http://www.pathology.leedsth.nhs.uk/dnn_bilm/Investigationprotocols/Pituitaryprotocols/TRHTest.aspx). TRH is not commercially available in the U.S. Future studies comparing the results of FTI and TRH stimulation test, in patients with suspected central hypothyroidism, would be of great interest and will hopefully be the subject of future research in areas of the world where TRH is commercially available.

One patient (Case 2) was receiving octreotide LAR therapy when the diagnosis of central hypothyroidism was made. A reduction in TSH secretion with somatostatin analogue therapy has been reported in the literature [9, 10]. While this is usually not clinically relevant (i.e., it does not usually cause overt central hypothyroidism), certainly it is possible that the therapy may have played a role in the development of central hypothyroidism in this patient.

It is unclear why measurement of serum FTI compared to FT4 may provide additional case detection in patients with central hypothyroidism. One may speculate that the normal reference range for serum FT4 may need to be reexamined in a larger reference population. It is important to note that while measurement of FTI may provide us with a better tool to identify additional cases of central hypothyroidism (higher sensitivity), in our experience it is not uncommon to get low FTI values between 5 and 6 (normal range: 6–11 *μ*g/dL) in patients without pituitary disorders (lower specificity). Therefore, it is very important to interpret thyroid function studies in the appropriate clinical context, taking into consideration the presence of symptoms and the status of other pituitary axes. This report documents our personal experience and may be assay dependent and needs to be further evaluated in future studies. Moreover, one must consider that free thyroxine assays have low precision at the lower end of the reference range and high interassay variability [11]. Clinicians should be familiar with the specific assay being utilized at their institution.

While this study reports an interesting observation, it is not without limitation. It is a retrospective study that only includes 4 subjects. Further prospective studies are needed to compare the two tests (FTI versus FT4). The goal of this report is to alert clinicians about the limitation of FT4 measurements in evaluating suspected cases of partial secondary hypothyroidism and that assessment of serum FTI may better allow for identification of such cases. Certainly one may argue that instead of measuring FTI the possibility of secondary hypothyroidism in the presence of low-normal free T4 in the appropriate clinical context should be taken into account and a trial of hormone replacement be considered. It is unknown whether the measurement of free T4 by some other method, such as equilibrium dialysis, would have assisted in identifying these patients as having central hypothyroidism. Future studies assessing serum FTI and FT4 in patients with new-onset central hypothyroidism and primary hypothyroidism would be helpful in further evaluating our observations.

It is also important to note that there are multiple ways of assessing the free thyroxine index, using T-uptake and T4, as was the case in our subjects, or using T4/TBG. Gupta et al. [8] compared these two methods, and the results of the regression analysis were similar, *r* = 0.94 for hypothyroid patients (primary hypothyroidism) and *r* = 0.89 for all patients. This high correlation indicates that the T4/TBG index may not provide any additional value over the standard FTI (T-uptake and T4) assessment; thus it is not normally used and will only increase the cost of testing. As TBG levels were not obtained on the subjects of this report, comparing these two methods in our subjects is not possible. Future research including assessment of the T4/TBG index in the evaluation of suspected cases of central hypothyroidism and a comparison of these values to that of the FTI (T-uptake and T4) would be of great interest.

Lastly, it is important to note that the reference ranges for the thyroid function studies included in this report were derived from the general population. It is possible that some of the subjects included in our report may have had circulating anti-TPO antibodies, and this could theoretically explain why the FT4 values were in the low-normal reference range (i.e., they may have an underlying recent-onset primary thyroid disorder). Unfortunately, as we were not suspecting a primary thyroid disorder in these subjects, given their clinical presentation, the anti-TPO status of the subjects was not assessed.

## 4. Conclusion

In the appropriate clinical setting, the measurement of serum FTI may provide additional case detection of central hypothyroidism when free T4 is in the low-normal range. Further prospective studies are needed to compare the use of FTI and FT4 in the evaluation of TSH axis in patients with pituitary disorders.

## Availability of Supporting Data

All data supporting the results of this paper are included in the paper. There is no additional unpublished data.

## Figures and Tables

**Table 1:** 

Pituitary disorder	TSH	T4	T4 uptake	FTI	FT4	FT3	T3	Albumin	Hormone deficiencies
	(0.4–5.5 *μ*U/mL)	(5–11 mcg/dL)	(0.7–1.2)	(6–11 mcg/dL)	(0.7–1.8 ng/dL)	(1.8–4.6 pg/dL)	(94–170 ng/dL)	(3.5–5.0 g/dL)	
Prolactinoma	0.922	4	1.06	3.8	0.7	2.1	88	4.2	Hypogonadism
Acromegaly	0.85	4.4	0.89	4.9	0.9	3	81	4.2	Panhypopituitarism
Macroadenoma (NF^*∗*^)	4.06	5.6	1.34	4.2	0.7	N/A	93	4.7	Hypogonadism
Macroadenoma (NF)	5.44	5.5	1.28	4.3	0.7	N/A	N/A	4.3	Panhypopituitarism

^*∗*^NF: nonfunctional.
